# In vitro processes alter the embryonic disc epigenome and transcriptome in the pre-implantation elongated bovine embryo^†^

**DOI:** 10.1093/biolre/ioaf095

**Published:** 2025-06-13

**Authors:** Thomas Behrens, Janaki Balasubramanian, Marilin Ivask, Monika Nõmm, Ants Kavak, Jan Bojsen-Møller Secher, Haja N Kadarmideen, Maria Belen Rabaglino

**Affiliations:** Department of Population Health Sciences, Faculty of Veterinary Medicine, Utrecht University, Utrecht, The Netherlands; Department of Population Health Sciences, Faculty of Veterinary Medicine, Utrecht University, Utrecht, The Netherlands; Institute of Veterinary Medicine and Animal Sciences, Estonian University of Life Sciences, Tartu, Estonia; Department of Veterinary Medicine, Faculty of Animal Sciences and Food Engineering, University of São Paulo, Pirassununga, Brazil; Institute of Veterinary Medicine and Animal Sciences, Estonian University of Life Sciences, Tartu, Estonia; Institute of Veterinary Medicine and Animal Sciences, Estonian University of Life Sciences, Tartu, Estonia; Department of Veterinary Clinical Sciences, University of Copenhagen, Copenhagen, Denmark; Department of Animal and Veterinary Sciences, Aarhus University, Tjele, Denmark; Department of Population Health Sciences, Faculty of Veterinary Medicine, Utrecht University, Utrecht, The Netherlands

**Keywords:** multi-omics, machine learning, transcriptomics, epigenomics, pregnancy, in vitro production, embryo competence index, cattle, artificial insemination, elongated embryo

## Abstract

The objective was to quantify the effect of in vitro procedures on the epigenome and transcriptome of the embryonic disc (ED) and extra-embryonic membranes (EEM) of day 15 in vitro produced (IVP) conceptuses compared to their in vivo (IVV) counterparts. IVP embryos (*n* = 7) were cultured serum-free until transfer at day 7, while IVV embryos (*n* = 9) were conceived through artificial insemination. Animals were flushed at day 15 of gestation, and sections of the ED and EEM underwent DNA and RNA extraction for whole-genome bisulfite or RNA sequencing. Raw fastq files were aligned to the ARS-UCD1.3 bovine genome. Processed data were integrated through a multi-omics approach based on machine learning to determine the key ontological terms that characterize each embryonic tissue lineage according to their methylome and transcriptome, followed by overrepresentation analyses (adjusted *P*-value < 0.05) of differentially methylated genes (DMG), differentially expressed genes (DEG), or genes that were both differentially methylated and differentially expressed in the ED or EEM of IVP compared to IVV conceptuses. Results demonstrated that identified critical ontological terms for the ED, such as somitogenesis, mesoderm formation, and gastrulation, were enriched among hypermethylated DMG, down-regulated DEG, and genes hypermethylated in the promoter and inhibited in expression in the ED of IVP embryos. Genes hypermethylated in the promoter and inhibited in expression in the EEM of IVP conceptuses were involved in epigenetic regulation. In conclusion, in vitro procedures alter the development of main lineage tissues in the pre-implantation embryo, even after interaction with the maternal environment.

## Teaser

The epigenome and transcriptome of embryonic disc and extra-embryonic membranes from elongated bovine conceptuses at day 15 indicate an abnormal development due to in vitro procedures compared to contemporaneous conceptuses produced in vivo.

## Graphical Abstract

**Graphical Abstract:**
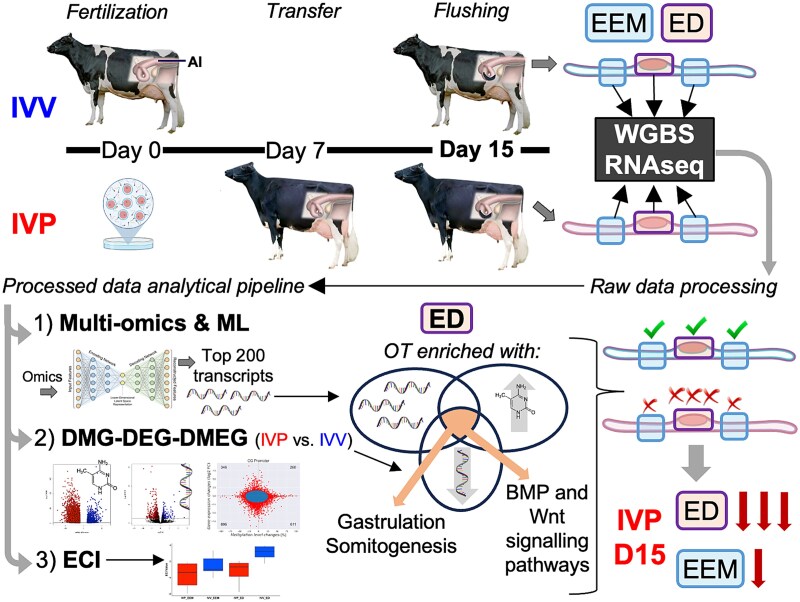

In vivo (IVV) embryos (*n* = 9) were conceived through artificial insemination, while IVP embryos (*n* = 7) were cultured in serum-free medium until transfer at day 7. Animals were flushed at day 15 of gestation, and sections of the embryonic disc (ED) and extra-embryonic membranes (EEM) were subjected to whole-genome bisulfite sequencing (WGBS) or RNA sequencing for epigenomics or transcriptomics, respectively. The processed data were analyzed in three steps: first, a multi-omics approach based on machine learning was applied to determine the top 200 transcripts that characterize each tissue lineage. Next, differentially methylated genes (DMG), differentially expressed genes (DEG), or genes that were both differentially methylated and differentially expressed (DMEG) in the ED or EEM of IVP conceptuses compared to their IVV counterparts were identified. Finally, an embryonic competence index (ECI) for each tissue and condition was estimated. Several ontological terms enriched by the characterizing transcripts were also associated with hypermethylated DMG and down-regulated DMEG, mainly in the ED. The high ECI value of the ED in the IVV embryos was reduced in the IVP conceptuses. In conclusion, in vitro processes alter the development of the main lineage tissues in the pre-implantation conceptus at day 15 of gestation, with a more pronounced effect in the ED than in the EEM.

## Introduction

A major goal for the future of humankind is to end hunger while promoting sustainable agriculture (https://sdgs.un.org/goals). Sustainable meat and milk production could be achieved by enhancing resilience and productivity traits while minimizing the impact on climate change. In cattle, assisted reproductive technologies, particularly those involving embryo production, are the most promising tools for attaining this end [1]. Embryos produced either in vitro (IVP) or in vivo (IVV) can be employed to disseminate the superior genetics of the female and accelerate genetic gain [2], be themselves subjected to genomic selection [3, 4], and to enhance the fertility of animals suffering from heat or metabolic stress [5]. The transfer of IVP embryos carries the advantages of diminishing the economic costs for embryo production, the opportunity to obtain genetic material from very young females, and “recycling” genetic information (oocytes) from animals sent to the food chain. For these and other reasons, since 2016, the transfer of IVP embryos has passed, and currently far exceeds, the transfer of IVV bovine embryos [6].

However, the IVP technology comes with drawbacks. For instance, embryonic genome activation (EGA), which occurs between the 8- and 16-cell stages, involves the transition of genomic control from the maternal genome to the embryonic genome [7, 8]. This activation in the embryo takes place in a culture medium instead of the natural maternal oviduct environment, which influences its transcriptome [9, 10] and potentially diminishes the embryo’s ability for pregnancy establishment. In addition, the stress caused by the environment and manipulations can induce epigenetic modifications in the embryo genome, such as DNA methylation (one of the most stable mechanisms), leading to permanent alterations in their transcriptome [11]. In fact, calves conceived in vitro still present modifications in the epigenome and transcriptome of several organs [12, 13], or phenotypically [14], when compared to calves conceived in vivo. Nevertheless, one of the main issues associated with IVP embryos is that their competence, or ability to sustain a pregnancy, is more compromised than an IVV embryo. According to a review published in 2019, which overviewed studies that compared IVP pregnancies with pregnancies using IVV embryos generated by superovulation, the pregnancy rate ranged between 29.5% and 54.3% (average 40.1%) after IVP embryo transfer and between 41.5% and 90% (average 64.1%) after IVV embryo transfer. Therefore, pregnancy rates for IVP embryos were around 25% lower than IVV embryos, with most pregnancy losses occurring between day 18 and 30 of gestation [15].

To shed light on the pregnancy loss etiology of IVP embryos, the main developmental events after transfer on day 7 should be reviewed. Once the embryo is deposited into the uterus, usually at the blastocyst stage, it induces local endometrial genes to interact with the intrauterine environment as soon after its transfer, even before hatching [16, 17]. Next, the blastocyst hatches from the zona pellucida at around day 8, coincident with the differentiation of the inner cell mass (ICM) into two cell lineages [18]: the epiblast, which will form the embryonic disc (ED), and lastly, the fetus, and the hypoblast, which, together with the trophectoderm (TE), constitutes the extra-embryonic membranes (EEM) that will originate the placenta. EEM proliferation initiates the conceptus elongation at around day 13, becoming filamentous by day 16–17 [19], when cells of the TE start attaching to the uterine epithelium, to begin implantation at around day 19 [20]. The elongation process is required to increase the number of TE cells that secrete interferon tau, the factor involved in maternal recognition of pregnancy in ruminants, to abolish the uterine luteolytic mechanism and ensure the maintenance of a functional corpus luteum for progesterone production [21].

Death of the IVP embryo after this period suggests that the elongation process is impaired, likely because of molecular alterations induced by the in vitro process. Indeed, we and others have shown that the epigenome [22], transcriptome [23, 24], or both molecular profiles [25] are altered in IVP conceptuses compared to IVV ones. However, these studies were done considering the whole embryo or only the TE layer, and therefore, the consequences of the in vitro process on the epigenomic and transcriptomic signatures of each tissue lineage are unknown. Since the in vitro procedures introduce genome-wide epigenetic changes, which, in turn, regulate gene transcription from early in development [26], we hypothesized that the IVP conceptus would show an impairment of critical processes belonging to the ED or EEM, leading to higher pregnancy losses. The objective of the current study was to quantify the effect of the in vitro procedures on the epigenome and transcriptome of ED and EEM of day 15 IVP conceptuses compared to IVV ones. To accomplish this objective, our analytical approach consisted of using multi-omics and machine learning to identify the key ontological terms characterizing each embryonic tissue lineage according to their methylome and transcriptome, followed by the determination of differentially methylated genes (DMG), differentially expressed genes (DEG), or differentially methylated and differentially expressed genes (DMEG), in the ED or EEM from IVP compared to IVV conceptuses. Additionally, we measured the developmental competence of each tissue lineage under each condition using the embryonic competence index (ECI), which measures the ability of the embryo to survive based on the expression of biomarker genes [27].

## Materials and methods

### Ethics

Animal procedures were approved by the Committee for Conducting Animal Experiments at the Ministry of Regional Affairs and Agriculture, Estonia (approval number 143 from 15.05.2019).

### In vitro embryo production

IVF Bioscience media and protocols were used for in vitro embryo production, unless otherwise stated.

Briefly, bovine ovaries (Holstein, *Bos taurus*) were collected from the slaughterhouse and the cumulus–oocyte complexes (COC) were aspirated from follicles with a diameter of 2–8 mm. The selected quality grade 1 COC (IETS manual, 5th edition, 2022) were matured in groups of 50 oocytes in 500 μL of BO-IVM medium in four-well plates (Nunc, Thermo Fisher Scientific) at 38.5°C with 6% CO_2_ in humidified atmospheric air for 22–24 h.

The matured oocytes were fertilized with frozen–thawed semen from a single Holstein (*B. taurus*) bull. The semen was washed twice with BO-SemenPrep and diluted to a final concentration of 2 × 10^6^ sperm/mL. Oocytes and sperm were co-incubated in groups of 50 in 500 μL of BO-IVF medium in four-well plates at 38.5°C with 6% CO_2_ in humidified atmospheric air for 18–20 h.

Cumulus cells were removed from the presumptive zygotes by vortexing, and the denuded embryos were transferred into 500 μL of BO-IVC medium under oil overlay. The embryos were cultured at 38.5°C, 6% CO_2_, 6% O_2_, and 88% N_2_ with humidified air for 7 days.

On the seventh day of in vitro culture (IVC), the embryos were checked for blastocyst formation. The blastocyst rate was 23.1% (106/458). The selected blastocysts were washed twice with BoviHold medium (Minitube) and transferred to synchronized recipient cows.

### In vitro embryo transfer

Recipient cows (Holstein, *B. taurus*, *n* = 2) were treated twice, at a 14-day interval, with a 25 mg intramuscular (IM) injection of prostaglandin F2α (PGF_2_α) (Dinolytic, 12.5 mg/mL, Zoetis Belgium SA). If the animals were in estrus on the fourth day after the second PGF2α injection, they were given gonadotropin-releasing hormone (GnRH) (lecirelin acetate, 25 μg/mL, Dalmarelin, Fatro S.p.A.) 2 mL per injection IM to ensure ovulation according to the manufacturer’s instructions.

Seven days after estrus and GnRH administration, fresh IVP embryos (*n* = 85) were transferred using a non-surgical technique. Prior to embryo transfer, recipients received low sacral epidural anesthesia with xylazine (Xylapan, Vetoquinol Biowet Sp. z o.o) at 0.05 mg/kg body weight diluted in 5 mL of 0.9% NaCl solution.

### In vivo embryo production

For in vivo embryo production, cows (Holstein, *B. taurus*, *n* = 2) were treated twice, at a 14-day interval, with a 25 mg IM injection of PGF_2_α (Dinolytic, 12.5 mg/mL, Zoetis Belgium SA). Estrus was detected on the third day after the second PGF_2_α injection. On day 10–13 after detecting estrus, 630 mg of follicle-stimulating hormone (FSH) (Folltropin-V, Bioniche Animal Health, Canada) was administered in eight tapering (105, 87.5, 70, and 52.5 mg) doses twice daily (AM:PM). On day 12, 2 mL of PGF_2_α (Dinolytic, 12.5 mg/mL, Zoetis Belgium SA) was administered twice daily (AM:PM). On the morning of day 14, 2 mL of GnRH (lecirelin acetate, 25 μg/mL, Dalmarelin, Fatro S.p.A.) was injected and artificial insemination (AI) was performed twice (AM:PM). Donors were inseminated with the frozen–thawed semen of the same Holstein bull that was also used for in vitro embryo production.

### Recovery of day 15 conceptuses

**Figure 1:**
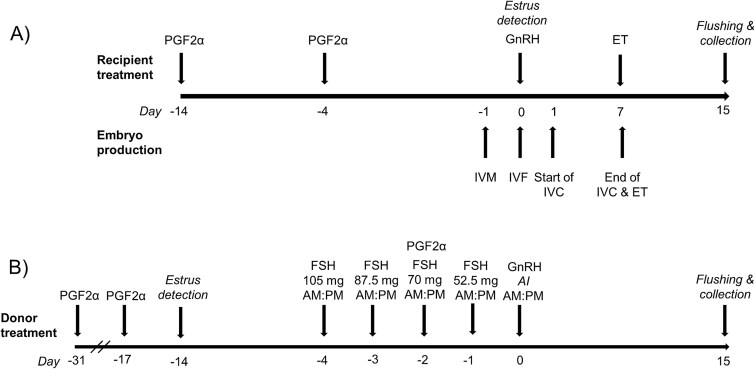
Diagram of the hormonal treatments performed in the (A) recipients, together with the laboratory procedures, for the generation and collection of day 15 conceptuses produced in vitro or (B) donors for the generation and collection of day 15 conceptuses produced in vivo.

The diagram of the hormonal treatments performed on the animals in each group is shown in Figure 1. Eight days after transfer of IVP embryos or 15 days after AI on superovulated cows, the embryos were non-surgically flushed out of the uterine horns using an 18 FR Foley catheter (Minitube) and BoviFlush (Minitube) solution. Flushing of embryos was performed under low sacral epidural anesthesia using xylazine (0.05 mg/kg, Xylapan, Vetoquinol Biowet Sp. z o.o) diluted in 5 mL of 0.9% NaCl saline.

The recovered day 15 embryos (*n* = 18 for IVV and *n* = 38 for IVP) were washed twice with BoviFlush (Minitube) and held in BoviHold medium (Minitube). The embryos were gently checked for the presence of ED and washed twice with 0.1% PVA and PBS medium. The ED was found in 94.4% (17/18) of the IVV embryos and in 55.3% of the IVP embryos (21/38). Embryos without ED were discarded. Embryo lengths were estimated under the microscope, using the ImageJ software. IVP embryos were shorter than IVV embryos (4.53 ± 3.63 vs 13.03 ± 8.04 mm, *P* = 0.01), according to an analysis of variance using the GLM procedure of the Statistical Analysis System v 9.4 (SAS Inst. Inc, Cary NC). Representative pictures of the recovered embryos and estimated lengths are shown in Figure S1.

The selected embryos were cut into three pieces. The ED and two pieces of surrounding EEM were snap-frozen in liquid nitrogen until genomic DNA (gDNA) or messenger RNA (mRNA) extraction and sequencing. The final sample size used in the bioinformatic analyses was, for the EEM, *n* = 6, and *n* = 8 for IVP and IVV embryos, respectively, while, for the ED, it was *n* = 7 and *n* = 9 for IVP and IVV embryos, respectively.

### DNA/RNA extraction, library preparation, and sequencing

The frozen samples were sent to BGI Tech Solutions (Hong Kong) Co., Ltd., which performed the gDNA and mRNA extraction, quality control, library preparation, and sequencing. The gDNA samples were subjected to WGBS, through bisulfite library preparation and PE100 sequencing with 45Gb clean data per sample on DNBseq. RNAseq was run in the RNA samples through non-stranded & polyA-selected mRNA library preparation and PE100 sequencing with 5Gb clean data per sample on DNBseq. For both techniques, after library preparation and sequencing, raw data with adapter sequences or low-quality sequences were filtered to remove contamination and obtain valid data. This step was completed by the SOAPnuke software [28]. Clean raw data were generated in the FASTQ format.

### Bioinformatic analysis

The WGBS clean reads were aligned to the ARS-UCD 1.3 bovine reference genome using the Bismark Bisulifte Read Marker (v 0.22.3) [29]. All covered cytosines were used for calculation of global CpG methylation level in Bismark using the following formula: percent global methylation = (number of methylated cytosines/total number of cytosines) × 100.

The RNAseq reads were mapped to the bovine reference genome (bosTau 9) with the STAR aligner (V 2.7.0b) [30], generating the genome index with the ARS-UCD1.3 assembly. Read counts were estimated at the gene level, and the counting was done with featureCounts [31], part of the Subread software (V 2.0.3).

### Identification of features characterizing each tissue lineage

A multimodal autoencoder consisting of two modules (encoder and decoder) was employed to extract the main features of each omics dataset, as detailed by Li et al. [32] for unsupervised multi-omics integration. The epigenome and transcriptome matrices were used as input data, using the default parameters. The analysis was performed with the MoGCN software for the Python environment [32]. The EntrezIDs corresponding to the top 200 transcripts for the ED and EEM were identified and subjected to overrepresentation analysis using ShinyGO 0.77 [33]; determining the enriched ontological terms as those with a false discovery rate (FDR) < 0.05.

### Determination of differentially methylated cytosines, differentially methylated genes, or differentially expressed genes on each tissue lineage

All the following analyses were performed with packages for the R software.

Differentially methylated cytosines (DMC) were determined with Methylkit [34]. To reduce the loss of data from merging across samples, a tiling function was used first to separate the genome into adjacent, 1000 bp tiles in which all methylated and unmethylated cytosines were given equal weight. Regions with a read coverage less than 10 or above the 99.9th percentile were removed to account for low coverage and polymerase chain reaction duplication bias, respectively, and then normalized with default parameters. Samples were merged by identifying regions common to at least seven out of eight samples per group. Regions with little or no variation, identified by a standard deviation of less than 2%, were filtered out. We used all remaining regions associated to a known chromosome for differential methylation calculations.

The log odds ratio of the methylation proportion *P_i_*, for a given sample *i*, at each region was modelled using a logistic regression, testing against the null hypothesis that the treatment, *T_i_*, had no effect on the methylation proportion. This model is shown below, in which *β_0_* represents the log odds of IVV samples and *β_1_* the log odds ratio between IVV and IVP. This model corrected for both the effect of the sex of the embryo and its morphology as covariates with associated parameters *α_1_* and *α_2_*.


$$ \log \left(\frac{P_i}{1-{P}_i}\right)={\beta}_0+{\beta}_1{T}_i+{\alpha}_1{Sex}_i+{\alpha}_2\,{Morphology}_i $$

Multiple testing was corrected for by converting *P*-values to *q*-values with the sliding linear model method. DMC were defined as those with a *q*-value of less than 0.01 or *P*-value <0.05 and a minimum absolute difference in methylation proportion of 0.2, which were annotated with the nearest transcription start site (TSS), and with their overlap with an exon, intron, intergenic, or CpG island using the Genomation package [35]. DMC within 1000 bp of a TSS were annotated as a promoter and those within 2000 bp of the end of a CpG island as a shore. The EntrezIDs corresponding to the annotated TSS related to DMC in a genic region (promoters, exons, and introns) of a protein-coding gene were defined as DMG.

Identification of DEG was performed through the DESeq2 [36], controlling for morphology with the sva package [37]. Gene expression counts were normalized by library size with DESeq2 methods, and the differential analysis was performed by fitting a logistic regression model to the gene counts, modeled by a negative binomial distribution, adjusting the *P*-values with Benjamini–Hochberg method. The Wald test statistic was employed to test for model significance. The DEG between IVP and IVV groups were defined as those with FDR < 0.05.

The EntrezIDs corresponding to DMG or to the DEG were subjected to overrepresentation analysis using ShinyGO 0.741 [33]; determining the enriched ontological terms as those with an FDR < 0.05.

### Promoter-level associations between changes in DNA methylation and gene expression between in vitro produced and in vivo embryos on each tissue lineage

This analysis was implemented with the MethGET software [38] for Python. Details about the method can be found in the corresponding publication. Briefly, the Cytosine reports generated by Bismark were converted to a CGmap format using a script provided by the software. Next, DNA methylation (CGmap files), gene expression (text files), and gene annotation (ARS-UCD1.3 assembly) data were input for the analysis. The overall correlations between DNA methylation level in the promoter region and gene expression for each tissue lineage were determined through the Pearson correlation coefficient, and genes differentially changing in DNA methylation (IVP – IVV) and gene expression (log2 IVP / IVV) between both conditions were identified through a Gaussian mixture model with a *P*-value <10^−3^. Given that methylation of the promoter region is associated with reduced gene transcription [39, 40], we defined DMEG as those that were hypermethylated in the promoter region and down-regulated in expression in the ED (or EEM) of IVP compared to IVV embryos.

The EntrezIDs corresponding to the DMEG were subjected to overrepresentation analysis using ShinyGO 0.741 [33], determining the enriched ontological terms as those with an FDR < 0.05.

### Determination of the embryonic competence index on each tissue lineage

The biomarker genes used to estimate the ECI were identified after applying machine learning approaches to transcriptomic data integrated from blastocysts and elongated embryos with different competences [27]. The goal of calculating the ECI in this study was to estimate the ability for pregnancy establishment of each tissue lineage and if they are affected by the in vitro procedure. The ECI is calculated using a regression equation that sums the products of each biomarker’s gene expression (normalized to a reference dataset) and its corresponding coefficient, along with an error term [41]. Results were compared by analysis of variance using the GLM procedure of the Statistical Analysis System v 9.4 (SAS Inst. Inc, Cary NC). The model included the tissue (ED or EEM), condition (IVP or IVV), and their interaction. Results are reported as the mean in the ECI values ± the standard deviation. Additionally, a linear correlation between the ECI values for ED and EEM and embryonic length was tested through the Pearson correlation coefficient (*R*). Significance was considered at *P*-value <0.05.

### Comparisons with other studies

The lists of up- and down-regulated DEG reported here overlapped with the DEG from other studies that also compared the transcriptome of bovine IVP versus IVV embryos. In one study, the DEG were determined on the EEM and chorion from day 18 and day 25, respectively, IVP conceptuses or IVV counterparts conceived through AI [42]. In the other study, the authors identified DEG in the TE and non-TE cells of IVP versus IVV blastocysts produced after superovulation treatment of the donor [43]. The EntrezIDs were used for comparisons. The test of independence (Pearson chi-square test) was employed to determine the relatedness (*P*-value <0.05) between up- or down-regulated DEG observed in IVP vs IVV embryos in our study and up- or down-regulated DEG in IVP vs IVV embryos in the two external studies.

## Results

### Profile of the genome-wide methylation and transcriptomic datasets

Each methylation call analyzed between 450 491 306 and 4 009 789 273 cytosines (mean = 3 157 731 364). Across all samples, 31.3% of these cytosines were in a CpG context and 0.8% in each of the CHG and CHH contexts.

After assigning cytosines to 1000 bp tiles, the number of tiles in a sample ranged between 104 852 and 1 911 566 (mean = 1 245 567). We identified 24 460 regions in the ED and 32 525 in the EEM that were common to at least six samples per treatment, to be used for differential methylation analysis.

mRNA reads generated after RNAseq resulted in a high proportion of reads with unique alignment to the *B. taurus* genome: 95.7% on average (94.1%–96.5%). The average proportions were alike among the tissue lineages (95.8% in ED and 95.5% in EEM) and among the conditions (95.5% for IVP 95.8% for IVV).

### Top selected features characterized each tissue lineage

**Figure 2:**
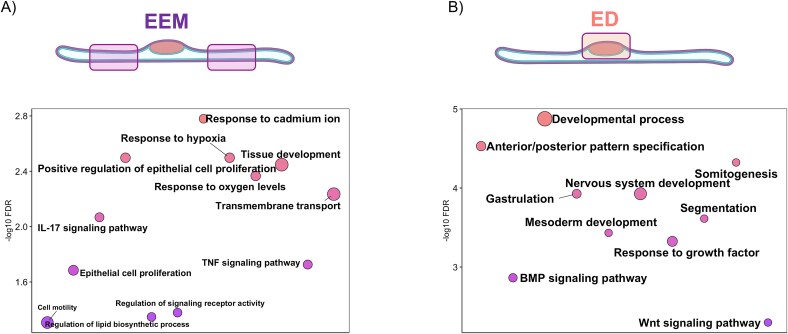
Ontological terms enriched among the top 200 transcripts characterizing the (A) extra-embryonic membranes (EEM) and (B) embryonic disc (ED) of day 15 elongated conceptuses produced in vitro or in vivo.

The autoencoder algorithm is a powerful multi-omics integration method for dimensionally reduction and extraction of important features from each omics layer [32]. The main ontological terms enriched among the top 200 transcripts for EEM were involved in response to oxygen levels, epithelial cell proliferation, and transmembrane transport, among others (Figure 2A, Table S1A). For the ED, the top 200 transcripts were enriched by ontological terms unique to this lineage, such as gastrulation, mesoderm formation, and somitogenesis (Figure 2B, Table S1B).

### Identification of differentially methylated genes and differentially expressed genes

Differential methylation analysis in the EEM tissue between IVP versus IVV embryos determined 5766 DMC associated with a gene, from which 4158 were annotated protein-coding genes. Of these DMG, 32.7% (1360) were hypermethylated and 67.3% (2798) were hypomethylated (Table S2A). For the ED, the number of DMC between IVP and IVV embryos was 6432, corresponding to 4668 DMG. From those DMG, 20.8% (970) were hypermethylated, while 79.2% (3698) were hypomethylated (Table S3A). The distribution of the association of these regions to promoter, exons, or introns is visualized in Figure S2.

**Figure 3:**
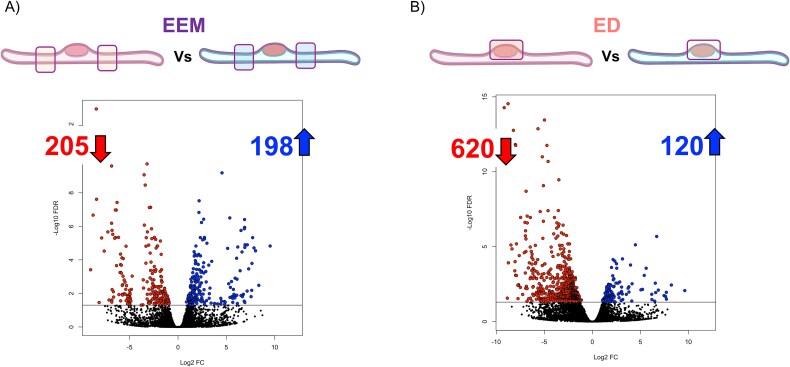
Differentially expressed genes (DEG) in the (A) extra-embryonic membranes (EEM) or (B) embryonic disc (ED) of day 15 elongated conceptuses produced in vitro compared to the in vivo counterparts. Blue and red dots in the volcano plots are DEG up- or down-regulated, respectively, in the in vitro produced embryo.

Differential expression analysis returned 403 and 740 DEG in the EEM and ED, respectively, between IVP and IVV conceptuses (Tables S4A and S5A). Figure 3 depicts the number of up- or down-regulated genes on each comparison.

### Functional analysis of differentially methylated genes and differentially expressed genes

**Figure 4:**
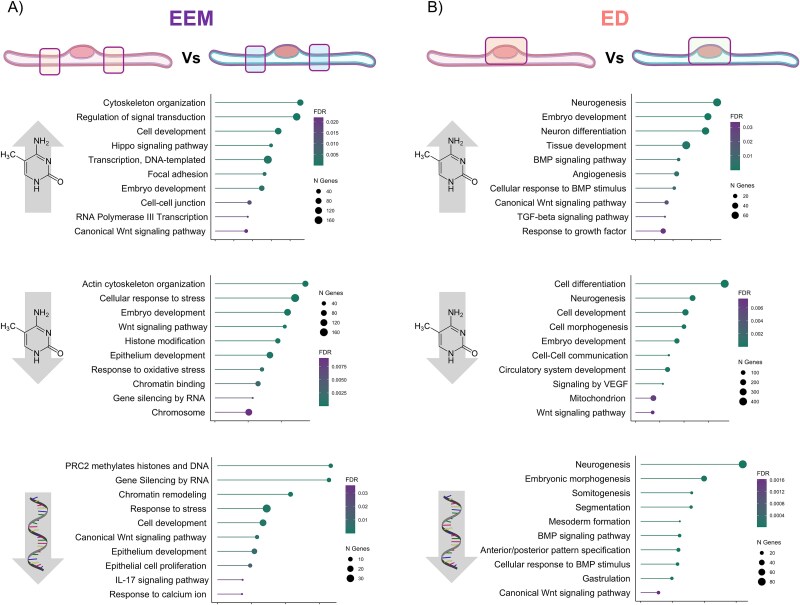
Main ontological terms enriched among hypermethylated genes (top), hypomethylated genes (middle), or down-regulated genes (bottom) in the (A) extra-embryonic membranes (EEM) or (B) embryonic disc (ED) of day 15 elongated conceptuses produced in vitro compared to the in vivo counterparts.

The overall lists of enriched ontological terms among the hyper- or hypo- DMG or up- or down-regulated DEG on each tissue list are detailed in the Supplemental files (DMG in EEM: Table S2B; DMG in ED: Table S3B; DEG in EEM: Table S4B and DEG in ED: Table S5B). While there were very few ontological terms enriched among the up-regulated DEG in both EEM and ED, most of the DMG or down-regulated DEG were involved in developmental processes. Figure 4 details the most relevant ontological terms enriched among the hyper- or hypo-DMG or down-regulated DEG. In the EEM, terms related to epigenetics modifications, such as chromatin binding or remodeling and histone modification, were enriched among the down-regulated DEG. Notoriously, for the ED, ontological terms critical for ED development were enriched among hypermethylated DMG and down-regulated DEG.

**Figure 5:**
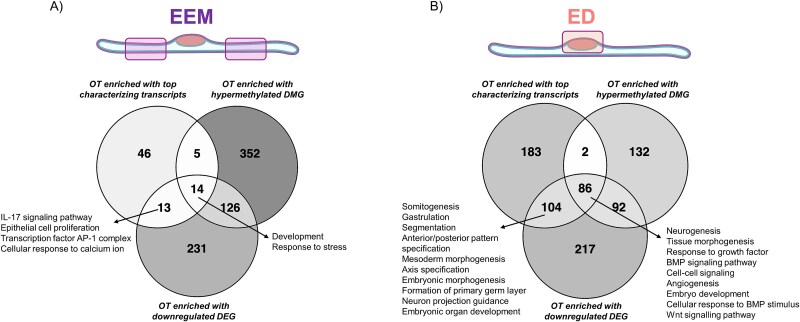
Overlap of enriched ontological terms among the top characterizing transcripts, hypermethylated genes, or down-regulated genes in the (A) extra-embryonic membranes (EEM) and (B) embryonic disc (ED) of day 15 elongated conceptuses produced in vitro or in vivo.

Furthermore, when comparing the enriched ontological terms related to the top 200 characterizing transcripts for each tissue lineage, hypermethylated DMG and down-regulated DEG (Figure 5), we found a strong overlap of terms in the ED (86 terms, 10.5% of the total) but to a lesser extent in the EEM (14 terms, 1.8% of the total). In the ED, key developmental processes such as Bone Morphogenetic Protein (BMP) and Wnt signaling pathways are enriched in both hypermethylated DMG and down-regulated DEG, while other ED exclusive processes, such as gastrulation, segmentation, axis specification, and mesoderm development are down-regulated in IVP embryos compared to IVV counterparts.

### Identification of differentially methylated genes on each tissue lineage

**Figure 6:**
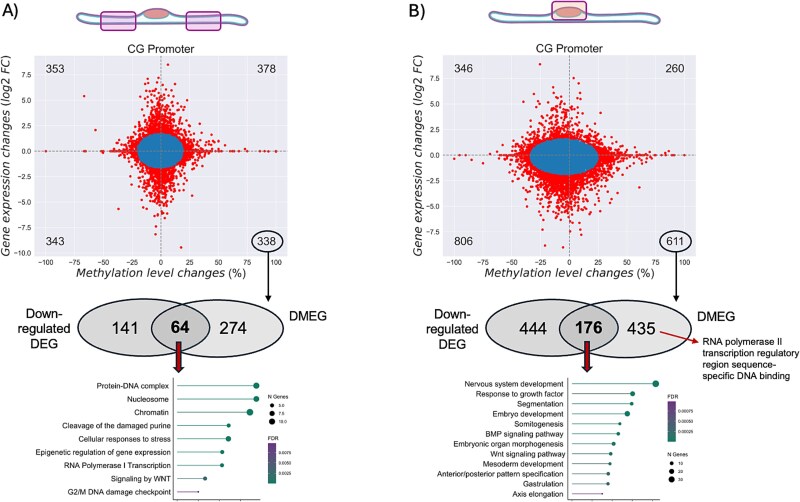
Promoter-level associations between changes in DNA methylation and gene expression in the (A) embryonic membranes (EEM) or (B) embryonic disc (ED). Red dots represent genes that are differentially methylated in their promoter regions and exhibit corresponding changes in mRNA expression (identified using a bivariate Gaussian mixture model; *P* < 10^−3^) in the EEM or ED of day 15 elongated conceptuses produced in vitro compared to their in vivo counterparts. Each quadrant of the plots displays the number of genes that are differentially methylated and differentially expressed. Genes that are both hypermethylated in their promoters and inhibited in expression were defined as differentially methylated and differentially expressed genes (DMEG; circled in the plots). Overlapping DMEG and down-regulated differentially expressed genes (DEG) are enriched for relevant ontological terms, as summarized in the corresponding lollipop plots.

The number of genes that were differentially methylated in their promoter regions and showed corresponding changes in mRNA expression was 1412 in the EEM and 2023 in the ED of IVP embryos compared to IVV counterparts (Figure 6, Tables S6A and S7A). The proportion of DMEG relative to all promoter-methylated genes was 23.9% (338/1412) in the EEM and 30.2% (611/2023) in the ED.

This integrative analysis supports the findings described above from the individual omics datasets. In the EEM, DMEG overlapping with down-regulated DEG were primarily associated with transcription and epigenetic regulation of gene expression (Figure 6A; Table S6B). In contrast, in the ED, DMEG overlapped with down-regulated DEG enriched for ontological terms related to gastrulation, segmentation, axis specification, mesoderm formation, and signaling pathways such as BMP and Wnt (Figure 6B; Table S7B).

### Ability of the embryos to sustain a pregnancy according to the embryo competence index

**Figure 7:**
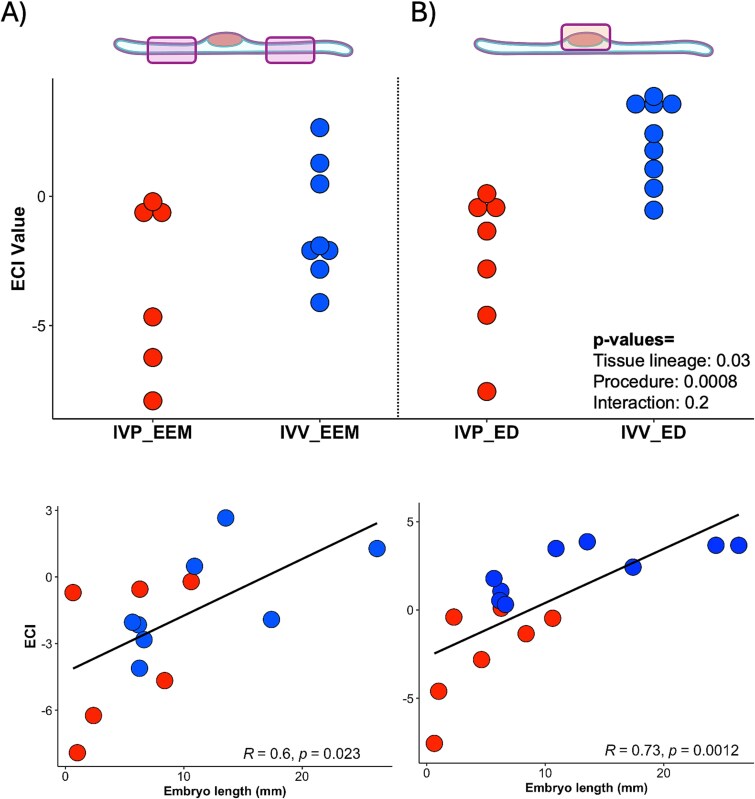
Embryonic competence index (ECI) values for the (A) embryonic membranes (EEM) or (B) embryonic disc (ED) of day 15 elongated conceptuses produced in vitro (IVP; red) or in vivo (IVV; blue), shown in the dotplots (top panels; the inset details the *P*-values for the effects of tissue lineage—EEM or ED, the procedure—IVP or IVV, and the interaction on the ECI values) or correlated with embryo length (bottom panels; the insets depict the Pearson correlation coefficient—*R*—and corresponding *P*-value).

The ECI values were affected by the procedure since IVP samples had a lower ECI value than IVV samples (−2.9 ± 2.9 vs 0.7 ± 2.5 for IVP and IVV, respectively) and by the tissue lineage, since the EEM showed a lower ECI than the ED (−2.1 ± 2.9 vs 0.2 ± 3.2 for EEM and ED, respectively). There was no interaction between the tissue lineage and the procedure, since the in vitro process lowered the ECI values in both tissue lineages (−3.4 ± 3.3 for EEM in IVP; −1.8 ± 2.3 for EEM in IVV; −2.4 ± 2.8 for ED in IVP and 2.2 ± 1.6 for ED in IVV; Figure 7A), suggesting that at day 15, IVP conceptuses were less likely to sustain a pregnancy than the IVV embryos. Furthermore, the ECI values for both EEM and ED were positively correlated with embryonic length (Figure 7B). As mentioned, IVP embryos were significantly shorter than the IVV counterparts (4.53 ± 3.63 vs 13.03 ± 8.04 mm).

### Overlap between differentially expressed genes in our study and differentially expressed genes determined in external studies

**Figure 8:**
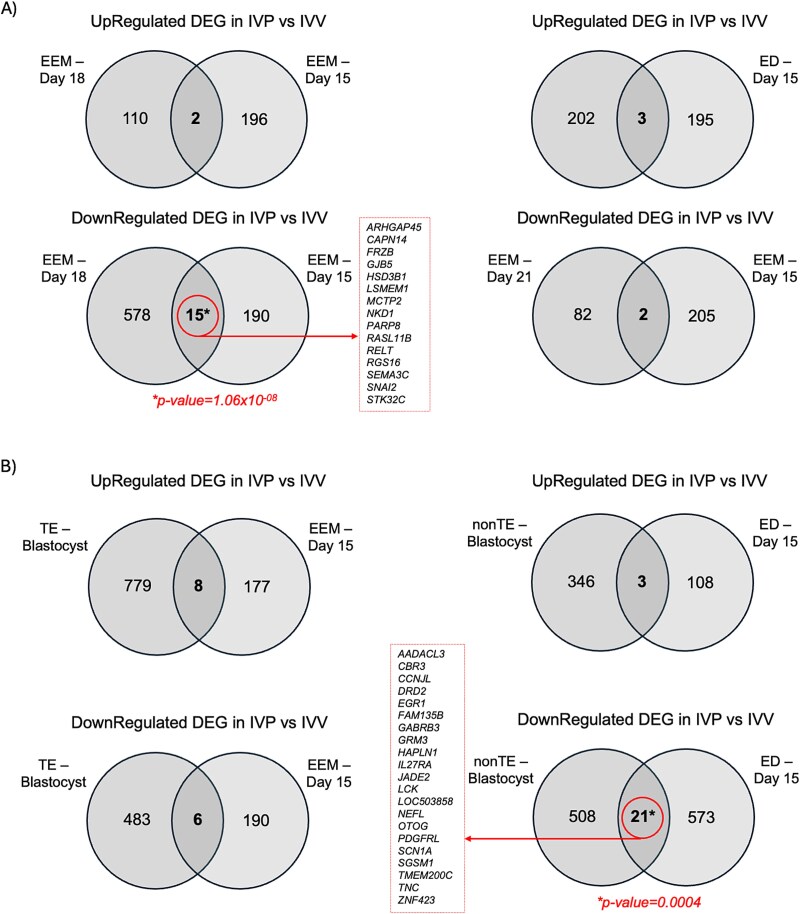
Overlap between up- and down-regulated differentially expressed genes (DEG) in the (A) extra-embryonic membranes (EEM) or chorion of in vitro (IVP) vs in vivo (IVV) produced day 18 and Day 21 conceptuses, as determined by Biase et al. [42] or in the (B) trophectoderm (TE) and non-TE cells of IVP vs IVV blastocysts, as determined by Ming et al. [43] with DEG in the EEM or embryonic disc (ED) of IVP vs IVV day 15 conceptuses, as we identified in this study.

There was no association between DEG determined for the Day 21 and Day 15 IVP vs IVV conceptuses. However, 15 down-regulated genes significantly overlapped between Day 18 and Day 15 IVP vs IVV conceptuses (Figure 8A). Three of these genes enriched for canonical Wnt signaling pathway (*FRZB, NKD1, SNAI2)*.

When the list of up- or down-regulated DEG in the TE and non-TE cells of IVP versus IVV blastocysts and in the EEM or ED of IVP versus IVV day 15 conceptuses were compared, there was a significant association of 21 down-regulated DEG in the non-TE cells of IVP blastocysts and in the ED of IVP day 15 conceptuses (Figure 8B). A functional analysis of these 21 genes revealed that 9 of them were involved in system development (*TNC, EGR1, DRD2, LCK, GABRB3, IL27RA, ZNF423, JADE2, NEFL*), while 5 of them were in neurogenesis and neuron projection (*DRD2, GABRB3, SCN1A, GRM3, NEFL*).

## Discussion

Higher pregnancy losses after the transfer of an IVP embryo are a negative consequence of this technology, for which the underlying mechanisms are multi-factorial and still not completely elucidated. Previous evidence suggests that IVP embryos have a compromised ability to trigger the appropriate molecular interaction with the maternal endometrium leading to attachment, and therefore, deregulation in conceptus–maternal communication can lead to embryonic death [42, 44, 45]. However, it is still unclear to what extent the main lineage tissues in the embryo are affected by the in vitro process after interaction with the maternal environment. Given that elongation of the IVP embryos is impaired, leading to IVP conceptuses of smaller size [23, 46], and differential DNA methylation was found to be prominent at intragenic sequences within the TE of IVP and IVV day 17 conceptuses [22], the EEM should be subjected to developmental errors in the IVP embryo, as we reported after a multi-omics metanalysis [25]. However, in vitro procedures introduce epigenomics changes early in development [26], even if the embryo was exposed to in vitro processes only during the period of EGA [47]. Therefore, both main tissue lineages would still be impacted in the peri-implantation period. To shed light on this aspect, we first applied a machine learning method (modified autoencoder) [32] to integrate both omics datasets and define the top transcripts characterizing each tissue. For analogy, the autoencoder is usually used in facial recognition, where after the input of different images of the face, the algorithm will extract the diagnostic features that allow reconstruction of the given face [48]. The tool proposed by MoGCN is an unsupervised method, i.e., we did not specify to which condition each sample belonged. The selected 200 transcripts for each tissue, out of ~17 000, were enriched for terms that might be occurring in EEM, such as response to oxygen levels or epithelial cell proliferation, but are known to be critical for the ED, such as gastrulation, somitogenesis, and pattern specification, confirming the effectiveness of the ED sampling. The next steps consisted of supervised differential methylation and differential expression analyses and promoter-level associations between methylation and gene expression changes, followed by an overrepresentation analysis of biological terms among the identified DMG, DEG, and DMEG.

Results from the above steps demonstrated an inhibition of the key ontological terms in the ED of IVP embryos compared to the ED of IVV embryos, suggesting a developmental alteration of IVP conceptuses, even beyond the morphological differences we controlled for. Results from the ECI support this statement. The ECI was effectively tested in embryos at the blastocyst and elongated conceptus stages, and it was found to be positively correlated with the length of day 17 conceptuses [41], as also observed in the present study. IVV embryos have higher ECI than IVP embryos, in agreement with the shorter length on average of the IVP conceptuses. Additionally, the difference in the ECI for ED and EEM was hindered because of the IVP process. In other words, the ED transcriptome, which is already differentiated at day 16 in the bovine IVV embryo [49], should indicate a higher capacity to establish pregnancy than the EEM transcriptome at day 15 of gestation, but this competence was potentially diminished in the ED of IVP conceptuses.

Differences in lengths between IVP and IVV conceptuses might also be attributed to the progesterone produced by multiple corpora lutea in response to the superovulation treatment performed in the IVV embryo donors before insemination since high progesterone concentration in the first few weeks post-ovulation primes the endometrium and accelerates conceptus elongation [50, 51]. Nevertheless, the epigenomic and transcriptomic differences observed in this study suggest that the IVP process could have impacted the ED differentiation, and day 15 embryos were abnormal compared to the IVV counterparts.

Notably, several genes related to ED-specific processes, such as somitogenesis and gastrulation, and to development, such as the BMP and the canonical Wnt signaling pathway, were hypermethylated in the promoter and thereby silenced in the ED of the IVP conceptuses. The BMP signaling triggers the cascade of signals controlling self-organized patterning, being essential for gastrulation, as shown by knockout studies in mice [52]. A study done with human embryonic stem cells determined that signaling from the *BMP4* gene induces the Wnt signaling pathway to promote mesoderm differentiation [53]. Specifically, the canonical Wnt signaling pathway, mediated by Wnt proteins such as *WNT3, WNT1, and WNT5a* [54], is primordial for primitive streak formation [55]. In our study, *WNT5A* and *BMP4* were hypermethylated in the promoter and inhibited in expression in the ED of the IVP conceptuses compared to the IVV counterparts. Therefore, the in vitro process induces an epigenomic de-regulation of these and other important genes involved in the BMP and Wnt signaling pathway, which impair the gene expression and the coordinated development of linage tissues in the elongated embryo, at least at day 15 of gestation.

Thus, the next pivotal question is if an IVP embryo presents a molecular signature related to developmental delay only during the pre-implantation period or if it is a common consequence of the in vitro process, or in other words, induced by the lack of the oviductal environment, regardless of variables such as the IVP media, technical procedures, or parental characteristics. Recent results reported by two different groups support this last motion. Briefly, Biase et al. [42] measured the transcriptome on the EEM and endometrial tissues of pregnancies initiated by AI or in vitro, at days 18 and 25 of gestation, when TE cells have differentiated into the chorion to begin placentation [20]. The authors found that the in vitro process strongly altered the gene expression in both the conceptus and the endometrium and, interestingly, transcripts in the chorion of day 25 IVP conceptuses were present in the EEM of day 18 IVV conceptuses. The embryos were investigated at a later stage than in our study, i.e., during the period of attachment, and the results were limited to the EEM -or the chorion- and not the ED of the elongated embryos. However, deregulation of gene expression in the day 18 and day 25 IVP conceptuses suggests that the development of these embryos could have also been altered at day 15, as observed in our study and indicated by the significant overlap between down-regulated genes at day 18 and day 15 EEM of IVP embryos compared to IVV counterparts.

At the blastocyst stage, the in vitro processes notoriously retard the process of cell fate commitment to the ICM, as demonstrated by Ming et al. [43]. The authors conducted single-cell RNAseq analysis of bovine IVP or IVV blastocysts, and one of the aims was to determine how in vitro conditions can impact the transcriptome of the blastocyst. One of their conclusions was that developmental potential differences between IVV and IVP embryos were mainly contributed by ICM, which, as mentioned in the introduction, will originate a cell lineage that will differentiate into the ED. The authors found that DEG down-regulated in the non-TE cells of IVP embryos were involved in development and neurogenesis, which coincides with our findings. Furthermore, the significant overlap between the 21 down-regulated DEG in the non-TE cells of IVP blastocysts and in the ED of IVP day 15 conceptuses suggests that impairment of cell lineage differentiation is induced early in development in the IVP embryo, and it is carried over several days after the embryo is transferred and gets in contact with the uterine environment, potentially impeding implantation and pregnancy continuation.

A limitation of our study is that IVV embryos were conceived through AI, and thus, some molecular differences could have been attributed to the conceptus allogeneity. Also, although we performed bulk RNAseq on each main lineage tissue, the application of the single-cell RNAseq technology would have provided higher fidelity to discern the consequences of the in vitro procedure on each cell lineage. Nevertheless, the application of multi-omics in our study provided insights into the potential mechanisms involved in pregnancy losses of the IVP embryo.

In conclusion, our study on day 15 bovine embryos highlights the negative effect of the IVP technology on the developmental processes of both tissue lineages analyzed, this effect being more pronounced in the ED than in the EEM. Further studies can be aimed at stimulating the differentiation of the IVP embryo during in vitro culture to match its development with that of an IVV embryo. Optimization of the IVP technology is a current compromise that can positively impact the sustainability of food production derived from cattle.

## Supplementary Material

Figure_S1_ioaf095

Figure_S2_ioaf095

Supplementary_Tables_ioaf095

## Data Availability

Data are deposited in NCBI’s Gene Expression Omnibus and are accessible through GEO accession number GSE278243.
